# Detection of lymphocytic choriomeningitis virus (LCMV) in the common house mice (*Mus musculus*) in Italy: an underrecognized threat to human health

**DOI:** 10.1128/spectrum.00470-26

**Published:** 2026-06-30

**Authors:** Barbara Di Martino, Federica Di Profio, Serena Robetto, Maria Lucia Mandola, Chiara Nogarol, Giuseppina La Rosa, Elisabetta Suffredini, Fulvio Marsilio, Claudia Maria Trombetta, Francesco Pellegrini, Michele Camero, Vito Martella, Vittorio Sarchese

**Affiliations:** 1 Department of Veterinary Medicine, University of Teramo, Teramo, Italy; 2 Centro di Referenza Nazionale per le Malattie degli Animali Selvatici (CeRMAS), Istituto Zooprofilattico Sperimentale del Piemonte, della Liguria e della Valle d'Aosta, Italy.; 3 SS Virologia Specialistica, Istituto Zooprofilattico Sperimentale Piemonte, Liguria e Valle d'Aosta (IZSPLV), Italy; 4 National Center for Water Safety (CeNSia), Istituto Superiore di Sanità 9289, Rome, Italy; 5 Department of Food Safety, Nutrition and Veterinary Public Health, Istituto Superiore di Sanità 9289, Rome, Italy; 6 Department of Molecular and Developmental Medicine, University of Siena 9313, Siena, Italy; 7 Department of Veterinary Medicine, University of Bari Aldo Moro 9295, Bari, Italy; 8 Department of Pharmacology and Toxicology, University of Veterinary Medicine 72408, Budapest, Hungary; The University of Texas Medical Branch at Galveston, Galveston, Texas, USA

**Keywords:** viruses, mammarenaviruses, LCMV, mice, Italy

## Abstract

Lymphocytic choriomeningitis virus (LCMV) is a neglected zoonotic pathogen primarily transmitted by the house mouse (*Mus musculus*). Despite being an underestimated zoonotic threat in Europe, LCMV lacks comprehensive surveillance. In this study, we report the first molecular detection and full genomic characterization of LCMV in Italy. Between May and November 2021, 107 rodents were captured on livestock farms in the Piedmont Region and screened for arenaviruses using a pan-arenavirus RT-PCR assay. LCMV RNA was detected in three *Mus musculus* specimens (3.5%), all originating from two neighboring farms. Viral presence was confirmed by LCMV-specific qRT-PCR. Complete S and L genome segments were successfully obtained from one sample using a combination of RT-PCR and nanopore sequencing. Phylogenetic analyses placed the Italian strain within LCMV lineage I, clustering with strains previously detected in Europe. Host genetic analysis confirmed that infected mice belonged to the subspecies *Mus musculus domesticus*.

**IMPORTANCE:**

This study provides the first molecular evidence and complete genomic characterization of Lymphocytic choriomeningitis virus (LCMV) in Italy in its primary reservoir*, Mus musculus*. The identification of LCMV at the livestock-wildlife interface suggests a significant anthropozoonotic risk, particularly for farm workers. These findings emphasize the urgent necessity for integrated molecular surveillance and increased clinical awareness to better define the public health impact of LCMV in Italy.

## OBSERVATION

Lymphocytic choriomeningitis virus (LCMV) is a rodent-borne zoonotic virus belonging to the genus *Mammarenavirus* within the family *Arenaviridae,* which comprises enveloped viruses with a bisegmented RNA genome (1). The virus is globally distributed, reflecting the widespread presence of its primary reservoir, the common house mouse (*Mus musculus; M. musculus*), although other rodents may be occasionally infected. Humans acquire infection through direct or indirect exposure to infected rodents or their excreta (2). The clinical spectrum of LCMV infection in humans is highly variable, ranging from mild, self-limiting febrile illness to severe neurological disease, including meningitis and encephalitis. Infection during pregnancy is associated with serious teratogenic outcomes, such as congenital hydrocephalus and chorioretinitis (3). Nevertheless, owing to nonspecific clinical manifestations, limited physician awareness, suboptimal diagnostic tools, and inconsistent reporting, LCMV remains a largely underrecognized public health threat (4).

In Europe, serological studies reveal a sharp contrast between negligible baseline seroprevalence in the general population (<1%) (5) and localized endemicity, with rates exceeding 35% in specific cohorts and rural “hotspots” (6). In Italy, information on LCMV circulation among rodent reservoirs and humans is limited and relies almost exclusively on serological investigations. To date, no confirmed human cases have been reported. However, three longitudinal studies conducted in northern Italy have documented seropositivity to LCMV-like viruses among wild rodents and healthy human populations (7, 8), with evidence of increasing human exposure over time, from 2.4% in 2002 to 7.0% in 2015 (9). Together with the recent identification of a novel mammarenavirus in European hedgehogs in Italy (10), these observations motivated the implementation of a molecular surveillance program in rodents aimed at detecting arenaviruses, including LCMV, using both broad-range and specific primers.

**Fig 1:**
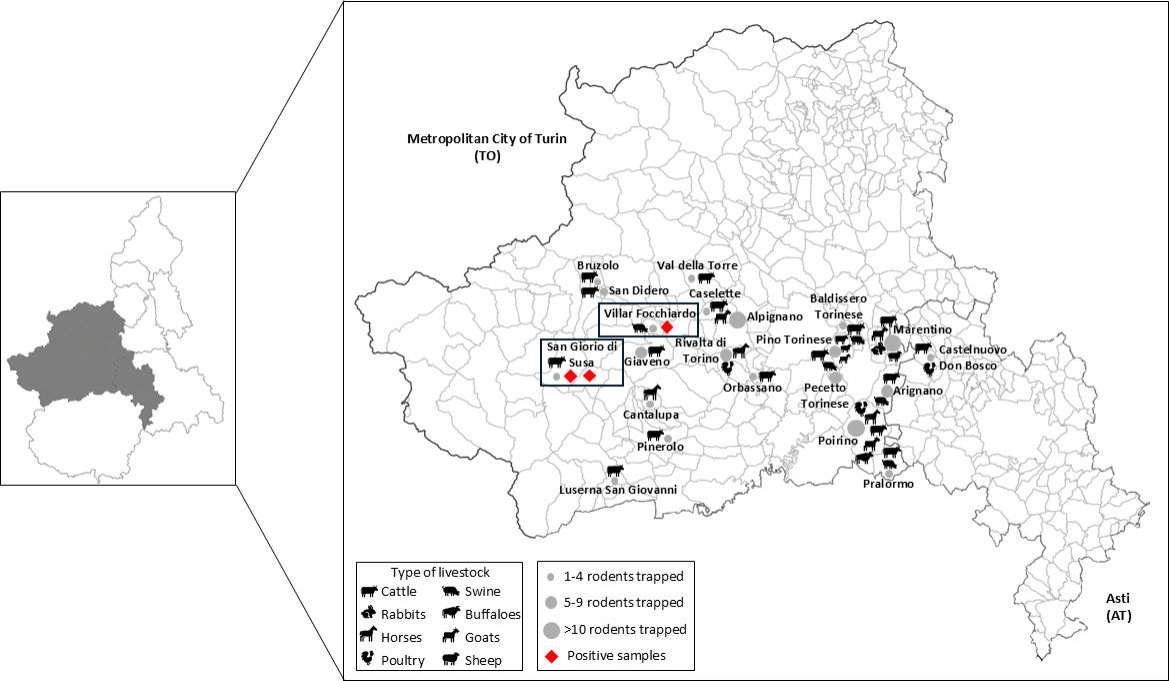
Geographic distribution of rodent sampling sites and livestock farms in the Piedmont Region (northwestern Italy), covering an area of 1,276.33 km² including the Susa Valley. Livestock types at each farm are represented by distinct symbols: cattle, swine, rabbits, buffaloes, horses, goats, poultry, and sheep. The map employs a proportional scaling method, in which the diameter of each grey circle correlates with the total number of rodents trapped at that specific location. LCMV-positive samples, indicated by red diamonds, were detected at two nearby farms located in Torino prefecture: San Giorio di Susa (cattle) and Villar Focchiardo (swine). Spatial data visualization was performed using Quantum GIS (QGIS) software, version 3.22. The map was obtained from d-maps.com, https://www.d-maps.com/carte.php?num_car=8263&lang=en.

Between May and November 2021, 107 rodents were trapped on 33 randomly selected farms across 21 municipalities (Table S1) in Piedmont Region (northwestern Italy) as part of a regional passive surveillance program performed by the Istituto Zooprofilattico Sperimentale del Piemonte, Liguria e Valle d’Aosta during pest-control activities routinely conducted on individual farms in compliance with Regulation (EC) No. 852/2004. Of the captured animals, 86 (80.4%) were identified as *M. musculus*, 6 (5.5%) as *Rattus norvegicus*, 6 (5.6%) as *Rattus rattus*, and 9 (8.4%) as belonging to the genus *Apodemus*. Liver, lung, and intestinal samples were collected and individually stored at −80°C before shipment to the Department of Veterinary Medicine of Teramo. Total RNA was extracted from 200 μL of tissue supernatant using the Direct-zol RNA MiniPrep Kit (Zymo Research). Initial molecular screening was performed using a pan-arenavirus reverse transcription (RT)-PCR assay targeting a 390-nucleotide (nt) conserved region within the polymerase domain of the large (L) gene (11). Viral RNA was detected in liver, lung, and intestinal samples from three house mice (IDs 54980-68/21, 54996-73/21, and 54999-74/21), corresponding to an overall prevalence of 3.5% (3/86) among *M. musculus*. All samples from other rodent species tested negative. The positive specimens were identified from two nearby farms in Torino prefecture, with two mice captured on a cattle farm in San Giorio di Susa and one captured on a swine farm in Villar Focchiardo (Fig. 1). This pattern may suggest localized viral circulation, potentially maintained by interconnected rodent populations. Pan-arenavirus amplicons were purified and subjected to Sanger sequencing. Pairwise sequence comparisons of the Italian sequences showed 93.6%–100% nucleotide identities to each other. FASTA analysis revealed the highest nucleotide identity (89.1%–89.7%) to reference LCMV sequences, while identities with other arenaviruses were below 60%. All tissue specimens were subsequently retested using a previously published real-time RT-PCR (qRT-PCR) assay targeting a conserved region of the nucleoprotein (NP) gene (12). Quantifiable LCMV RNA was detected exclusively in the samples previously identified as positive by the pan-arenavirus assay, with viral loads ranging from 2.5 × 10³ to 1.0 × 10⁴ genome copies/g. The highest viral titers were detected in liver samples (Table S2).

Attempts to reconstruct the complete viral genome were made for all the positive samples, applying a whole-segment one-step RT-PCR approach using a newly designed primer (Panhandle LCMV: 5′-TGCACAGGGGATCCTAGGC-3′) targeting the conserved panhandle regions present at both ends of the S and L genome segments (13, 14) (Fig. S1). Full-length amplification of the L segment was achieved by two subsequent semi-nested PCRs with pan-arenavirus primers (11), generating overlapping fragments of 4,016 nt and 3,607 nt, together covering the entire ~7.2 kb segment. Amplicons were purified, pooled equimolarly, and sequenced on a MinION Mk1C device using the Ligation Sequencing Kit (SQK-LSK110, Oxford Nanopore Technologies) and an FLO-MIN106 R9.4.1 flow cell. Raw reads were quality filtered, trimmed, and assembled by reference mapping using Minimap2 implemented in Geneious Prime v2024.0. The complete genome of strain LCMV/Mus/68/22/ITA (ID mouse 54980-68/21) was sequenced from a liver sample (GenBank: PX778817 [L segment] and PX778818 [S segment]). Due to panhandle primer design, terminal 5′ and 3′ sequences reflect primer composition and may not represent native polymorphisms. The L segment was 7,228 nt in length and comprised two open reading frames (ORFs) encoding the zinc-binding matrix protein (273 nt; 91 amino acids [aa]) and the viral RNA-dependent RNA polymerase (6,630 nt; 2,210 aa), separated by a 204-nt noncoding region. The S segment was 3,376 nt long and contained two ORFs encoding the glycoprotein precursor (1,497 nt; 499 aa) and the nucleoprotein (1,677 nt; 559 aa), separated by a 64-nt intergenic region.

**Fig 2:**
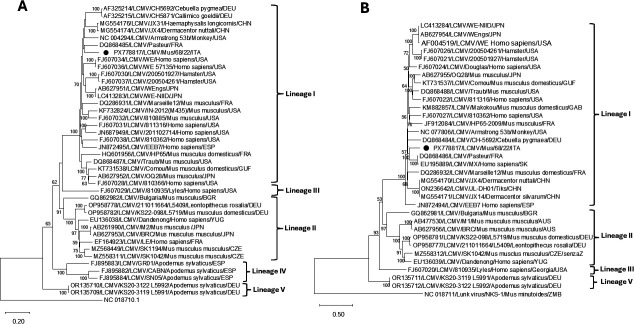
Phylogenetic analyses based on nucleotide sequences of the complete S (**A**) and L (**B**) segments of the LCMV strain identified in this study. The evolutionary history was inferred by using the Maximum Likelihood method and general time-reversible (GTR) model with a gamma distribution and invariable sites. Trees were elaborated by using the alignment of the full-length nucleotide S and L sequences of the LCMV Mus/68/22/ITA strain (black circle) generated in this study and the cognate S and L sequences of LCMV strains retrieved from GenBank (accession numbers shown). Initial tree(s) for the heuristic search were selected by choosing the tree with the superior log-likelihood between a Neighbor-Joining NJ tree and a Maximum Parsimony tree. A total of 1,000 bootstrap replicates were used to estimate the robustness of the individual nodes on the phylogenetic tree. Scale bar indicates number of nucleotide substitutions per site. In both trees, *Lunk virus* from *Mus minutoides* (Africa) was used as outgroup. Evolutionary analyses were conducted in MEGA12 (17).

Phylogenetic analyses were conducted using full-length sequences for both segments (Fig. 2). The S segment phylogenetic tree included sequences representing lineages I–V, whereas the L segment tree comprised sequences from the four available lineages (I, II, III, and V) (15, 16). Both segments of the LCMV/Mus/68/22/ITA genome clustered within lineage I, together with LCMV strains previously detected in humans and rodents in Europe (15, 16), with nt identities ranging from 83.1% to 86.3% for the S segment and from 80.0% to 81.8% for the L segment. Despite the robust assignment of the Italian strain to Lineage I, insufficient bootstrap support at internal nodes did not allow definitive conclusions regarding its specific geographic origin or evolutionary introduction route.

Previous studies have shown that LCMV lineages correlate more with the mouse host subspecies than geographic origin, with lineage I associated with the subspecies *M. musculus domesticus*, and lineage II with *M. musculus musculus* (16). To assess host-virus relationships, we analyzed a partial mitochondrial cytochrome *b* sequence by using previously designed degenerated primers (18). Phylogenetic analysis (Fig. S2) revealed that the three LCMV-positive mice identified in this study belonged to *M. musculus domesticus* subspecies widespread across western and southern Europe, including Italy.

In conclusion, we report the first molecular detection and genomic characterization of LCMV in Italy, filling a gap left by previous serologic studies. The detection of LCMV in livestock farms suggests possible human-rodent exposure among farm workers and underestimation of LCMV exposure in rural areas. Although no neurological or febrile illnesses were officially reported among local farm workers during the study period, these findings must be interpreted with caution as active human surveillance was not performed. Accordingly, our results emphasize the need for expanded surveillance of rodent reservoirs. Furthermore, given the risk of severe disease, especially in pregnant women and immunocompromised individuals, increased clinical awareness and monitoring are warranted. Finally, pan-arenavirus qualitative PCR and LCMV-specific quantitative PCR show comparable sensitivity, with the broad-range assay allowing the detection of diverse arenaviruses and early typing.

## Data Availability

The data that support the findings of this study are openly available in the GenBank database at https://www.ncbi.nlm.nih.gov/nucleotide/ under accession numbers: L segment no. PX778817; S segment no. PX778818.

## SUPPLEMENTAL MATERIAL

Fig. S1 spectrum.00470-26-s0001.tif Genome organization of LCMV/Mus/68/22/ITA and amplification strategy.

Fig. S2 spectrum.00470-26-s0002.tif Phylogenetic analysis performed on a partial fragment (385 nt) of the mitochondrial cytochrome b (Cytb) gene of *Mus musculus* spp.

Supplemental legends spectrum.00470-26-s0003.docx Legends for Fig. S1 and S2.

Table S1 spectrum.00470-26-s0004.docx Number of rodents captured between May and November 2021 on each of the 33 farms distributed across 21 municipalities in the Piedmont region (northwestern Italy).

Table S2 spectrum.00470-26-s0005.docx LCMV RNA viral loads detected by qRT-PCR in tissue samples of the three infected animals.

ASM does not own the copyrights to Supplemental Material that may be linked to, or accessed through, an article. The authors have granted ASM a non-exclusive, world-wide license to publish the Supplemental Material files. Please contact the corresponding author directly for reuse.
