# Supplementary material for: Detection of lymphocytic choriomeningitis virus (LCMV) in the common house mice (Mus musculus) in Italy: an underrecognized threat to human health
Source: Microbiol Spectr. 2026 Jun 30;14(8):e00470-26. doi: 10.1128/spectrum.00470-26 (PMC13436406; doi:10.1128/spectrum.00470-26)
Supplement: Supplementary file 1 [file spectrum.00470-26-s0003.docx]

Fig. S1. Genome organization of LCMV/Mus/68/22/ITA and amplification strategy. (A) The L segment (7,228 nt) encodes the Z protein (91 aa) at the 5′ end and the L polymerase protein (2,210 aa). Long RT-PCR was performed using a primer (5’-TGCACAGGGGATCCTAGGC-3′) targeting the conserved panhandle regions present at both ends of the S and L genome segments, to generate a large amplicon spanning the entire L sequence. To ensure complete coverage, an overlapping semi-nested PCR was applied using previously described pan-arenavirus primers (59A+, 59D+, 59G+, 54A-, 54D-) (9), producing two partially overlapping fragments (4,016 nt and 3,607 nt; 395-nt overlap). (B) The S segment (3,376 nt) encodes the GPC (499 aa) and NP (559 aa). It was amplified in a single long RT-PCR reaction using the panhandle primer, yielding an amplicon spanning the full S segment.

Fig. S2. Phylogenetic analysis performed on a partial fragment (385 nt) of the mitochondrial cytochrome b (*Cytb*) gene of *Mus musculus* spp. Tree was generated using Neighbor-Joining method based on the Tamura-Nei model and supplying statistical support with bootstrapping of 1000 replicates. Sequences generated in this study are indicated in black triangles. Evolutionary analyses were conducted in MEGA 12 (15).
