# Supplementary material for: Detection of lymphocytic choriomeningitis virus (LCMV) in the common house mice (Mus musculus) in Italy: an underrecognized threat to human health
Source: Microbiol Spectr. 2026 Jun 30;14(8):e00470-26. doi: 10.1128/spectrum.00470-26 (PMC13436406; doi:10.1128/spectrum.00470-26)
Supplement: Supplementary file 2 [file spectrum.00470-26-s0004.docx]

Table S1. Number of rodents captured between May and November 2021 on each of the 33 farms distributed across 21 municipalities in the Piedmont region (northwestern Italy).

| **Municipality (Province)** | **Farm ID** | **Farm type** | **Sampling date (month/year)** | **No. rodents trapped** | **No. *Mus musculus* trapped** | **No. positive *Mus musculus* (prevalence %)** | **Other rodent species trapped** | **No. positive of other rodent species (prevalence %)** |
| --- | --- | --- | --- | --- | --- | --- | --- | --- |
| Luserna San Giovanni (TO) | F1 | Cattle | 05/2021 | 3 | 3 | 0/3 (0%) |  |  |
| Giaveno (TO) | F2 | Cattle | 05/2021 | 6 | 6 | 0/6 (0%) |  |  |
|  | F3 | Cattle | 05/2021 | 1 |  |  | *Rattus norvegicus* (1) | 0/1 (0%) |
|  | F4 | Cattle | 05/2021 | 2 | 2 | 0/2 (0%) |  |  |
| Val della Torre (TO) | F5 | Cattle | 05/2021 | 2 | 2 | 0/2 (0%) |  |  |
|  | F6 | Cattle | 05/2021 | 1 | 1 | 0/1 (0%) |  |  |
| Alpignano (TO) | F7 | Goat | 05/2021 | 17 | 17 | 0/17 (0%) |  |  |
| Caselette (TO) | F8 | Cattle | 05/2021 | 2 | 2 | 0/2 (0%) |  |  |
| Castelnuovo Don Bosco (AT) | F9 | Poultry | 06/2021 | 3 | 3 | 0/3 (0%) |  |  |
|  | F10 | Cattle | 06/2021 | 1 | 1 | 0/1 (0%) |  |  |
| Baldissero Torinese (TO) | F11 | Swine | 06/2021 | 1 | 1 | 0/1 (0%) |  |  |
|  | F12 | Cattle | 06/2021 | 1 | 1 | 0/1 (0%) |  |  |
| Cantalupa (TO) | F13 | Horse | 07/2021 | 1 | 1 | 0/1 (0%) |  |  |
| Pinerolo (TO) | F14 | Cattle | 07/2021 | 1 | 1 | 0/1 (0%) |  |  |
| Rivalta di Torino (TO) | F15 | Horse | 10/2021 | 1 | 1 | 0/1 (0%) |  |  |
|  | F16 | Poultry | 10/2021 | 6 | 3 | 0/3 (0%) | *Apodemus spp.* (3) | 0/3 (0%) |
| Orbassano (TO) | F17 | Cattle | 10/2021 | 3 | 3 | 0/3 (0%) |  |  |
| Pralormo (TO) | F18 | Mixed Cattle and Swine | 10/2021 | 4 | 3 | 0/3 (0%) | *Rattus norvegicus* (1) | 0/1 (0%) |
| Poirino (TO) | F19 | Mixed Cattle, Goat, Horse | 10/2021 | 3 | 2 | 0/2 (0%) | *Rattus norvegicus* (1) | 0/1 (0%) |
|  | F20 | Mixed Buffalo and Poultry | 10/2021 | 6 | 5 | 0/5 (0%) | *Rattus norvegicus* (1) | 0/1 (0%) |
|  | F21 | Horse | 10/2021 | 1 | 1 | 0/1 (0%) |  |  |
| San Giorio di Susa (TO) | F22 | Cattle | 10/2021 | 2 | 2 | 2/2 (100%) |  |  |
| Bruzolo (TO) | F23 | Cattle | 10/2021 | 3 |  |  | *Rattus rattus* (3) | 0/3 (0%) |
| San Didero (TO) | F24 | Cattle | 10/2021 | 2 | 1 | 0/1 (0%) | *Apodemus spp (1)* | 0/1 (0%) |
| Villar Focchiardo (TO) | F25 | Swine | 10/2021 | 1 | 1 | 1/1 (100%) |  |  |
| Pecetto Torinese (TO) | F26 | Mixed Cattle and Swine | 11/2021 | 5 | 4 | 0/4 (0%) | *Apodemus spp.* (1) | 0/1 (0%) |
| Pino Torinese (TO) | F27 | Mixed Cattle and Sheep | 11/2021 | 1 |  |  | *Rattus rattus* (1) | 0/1 (0%) |
|  | F28 | Mixed Cattle, Sheep, Goat | 11/2021 | 5 | 2 | 0/2 (0%) | *Rattus rattus* (2) and *Rattus norvegicus* (1) | 0/3 (0%) |
| Marentino (TO) | F29 | Cattle | 11/2021 | 11 | 11 | 0/11 (0%) |  |  |
|  | F30 | Rabbit | 11/2021 | 4 |  |  | *Apodemus spp.* (4) | 0/4 (0%) |
|  | F31 |  |  |  |  |  |  |  |
|  | F33 | Mixed Cattle, Sheep, Goat | 11/2021 | 1 | 1 | 0/1 (0%) |  |  |
| Arignano (TO) | F31 | Cattle | 11/2021 | 4 | 4 | 0/4 (0%) |  |  |
|  | F32 | Swine | 11/2021 | 2 | 1 | 0/1 (0%) | *Rattus norvegicus* (1) | 0/1 (0%) |
| **Total** (21 municipalities) | **Total** (33 farms) |  |  | **Total** (107 rodents) | **Total** (86 *Mus musculus*) | **Total** Prevalence *Mus musculus* 3/86 (3.5%) | **Total** (21 Other Rodents); *Rattus norvegicus* (6), *Rattus rattus* (6) and *Apodemus spp*. (9) | **Total** Prevalence Other Rodents (0/21, 0%) |
