# Supplementary material for: Detection of lymphocytic choriomeningitis virus (LCMV) in the common house mice (Mus musculus) in Italy: an underrecognized threat to human health
Source: Microbiol Spectr. 2026 Jun 30;14(8):e00470-26. doi: 10.1128/spectrum.00470-26 (PMC13436406; doi:10.1128/spectrum.00470-26)
Supplement: Supplementary file 3 [file spectrum.00470-26-s0005.docx]

Table S2. LCMV RNA viral loads detected by qRT-PCR in tissue samples of the three infected animals

| ***Mus musculus* ID** | **Farm ID** | **Genome copies/g** | | |
| --- | --- | --- | --- | --- |
|  |  | **Liver** | **Lung** | **Intestine** |
| #54980-68/21 | F22 | 1.0 x 10^4^ | 3.0 x 10^3^ | - |
| #54996-73/21 |  | 4.2 x 10^3^ | 4.5 x 10^3^ | 3.5 x 10^3^ |
| #54999-74/21 | F25 | 2.5 x 10^3^ | 2.8 x 10^3^ | - |
